# Mitochondrial DNA Variants in Obesity

**DOI:** 10.1371/journal.pone.0094882

**Published:** 2014-05-02

**Authors:** Nadja Knoll, Ivonne Jarick, Anna-Lena Volckmar, Martin Klingenspor, Thomas Illig, Harald Grallert, Christian Gieger, Heinz-Erich Wichmann, Annette Peters, Susanna Wiegand, Heike Biebermann, Pamela Fischer-Posovszky, Martin Wabitsch, Henry Völzke, Matthias Nauck, Alexander Teumer, Dieter Rosskopf, Christian Rimmbach, Stefan Schreiber, Gunnar Jacobs, Wolfgang Lieb, Andre Franke, Johannes Hebebrand, Anke Hinney

**Affiliations:** 1 Department of Child and Adolescent Psychiatry, University of Duisburg-Essen, Essen, Germany; 2 Institute of Medical Biometry and Epidemiology, Philipps-University of Marburg, Marburg, Germany; 3 Molecular Nutritional Medicine, Technical University of Munich, Else Kröner-Fresenius Center, Freising-Weihenstephan, Germany; 4 Research Unit of Molecular Epidemiology, Helmholtz Center Munich – German Research Center for Environmental Health, Neuherberg, Germany; 5 Hannover Unified Biobank, Hannover Medical School, Hannover, Germany; 6 Institute of Epidemiology II, Helmholtz Center Munich – German Research Center for Environmental Health, Neuherberg, Germany; 7 German Center for Diabetes Research, Neuherberg, Germany; 8 Institute of Genetic Epidemiology, Helmholtz Center Munich – German Research Center for Environmental Health, Neuherberg, Germany; 9 Institute of Epidemiology I, Helmholtz Center Munich – German Research Center for Environmental Health, Neuherberg, Germany, Neuherberg, Germany; 10 Institute of Medical Informatics, Biometry, and Epidemiology, Chair of Epidemiology, Ludwig-Maximilians-Universität, Munich, Germany; 11 Munich University Hospital, Campus Grosshadern, Munich, Germany; 12 Institute of Experimental Pediatric Endocrinology, Charité Berlin, Germany; 13 Division of Pediatric Endocrinology and Diabetes, Department of Children and Adolescent Medicine, University of Ulm University Medical Center, Ulm, Germany; 14 Institute for Community Medicine, University Medicine Greifswald, Greifswald, Germany; 15 Institute for Clinical Chemistry and Laboratory Medicine, University Medicine Greifswald, Greifswald, Germany; 16 Institute for Pharmacology, University Medicine Greifswald, Greifswald, Greifswald, Germany; 17 Institute of Clinical Molecular Biology, Christian-Albrechts-University of Kiel, Kiel, Germany; 18 Institute of Epidemiology and Biobank popgen, Christian-Albrechts-University of Kiel, Kiel, Germany; Vanderbilt University Medical Center, United States of America

## Abstract

Heritability estimates for body mass index (BMI) variation are high. For mothers and their offspring higher BMI correlations have been described than for fathers. Variation(s) in the exclusively maternally inherited mitochondrial DNA (mtDNA) might contribute to this parental effect. Thirty-two to 40 mtDNA single nucleotide polymorphisms (SNPs) were available from genome-wide association study SNP arrays (Affymetrix 6.0). For discovery, we analyzed association in a case-control (CC) sample of 1,158 extremely obese children and adolescents and 435 lean adult controls. For independent confirmation, 7,014 population-based adults were analyzed as CC sample of n = 1,697 obese cases (BMI≥30 kg/m^2^) and n = 2,373 normal weight and lean controls (BMI<25 kg/m^2^). SNPs were analyzed as single SNPs and haplogroups determined by HaploGrep. Fisher's two-sided exact test was used for association testing. Moreover, the D-loop was re-sequenced (Sanger) in 192 extremely obese children and adolescents and 192 lean adult controls. Association testing of detected variants was performed using Fisher's two-sided exact test. For discovery, nominal association with obesity was found for the frequent allele G of m.8994G/A (rs28358887, p = 0.002) located in *ATP6*. Haplogroup W was nominally overrepresented in the controls (p = 0.039). These findings could not be confirmed independently. For two of the 252 identified D-loop variants nominal association was detected (m.16292C/T, p = 0.007, m.16189T/C, p = 0.048). Only eight controls carried the m.16292T allele, five of whom belonged to haplogroup W that was initially enriched among these controls. m.16189T/C might create an uninterrupted poly-C tract located near a regulatory element involved in replication of mtDNA. Though follow-up of some D-loop variants still is conceivable, our hypothesis of a contribution of variation in the exclusively maternally inherited mtDNA to the observed larger correlations for BMI between mothers and their offspring could not be substantiated by the findings of the present study.

## Introduction

Twin, adoption and family studies have shown that the heritability of the variation in body mass index (BMI) is high [1]–[4]. Genetic factors explain about 40% to 70% of the variance of the BMI [5]. Some family and adoption studies showed higher correlations in BMI between mothers and their offspring compared to fathers and their offspring e.g. [4, 6, 7]. In a study of 540 adult Danish adoptees, for instance, BMI correlation between biological mothers and offspring were 0.15 compared with 0.11 between biological fathers and offspring [4]. Among maternal half-brothers this effect was also shown as depicted by a two-fold higher BMI correlation (r = 0.21) compared to paternal half-brothers (r = 0.11) [8].

During the last years, several monogenic and polygenic forms of obesity have been elucidated (summarized in [5]). Large-scale genome-wide association studies (GWAS) and independent confirmation (up to 250,000 individuals in total) revealed several BMI and/or body weight associated loci. However, these studies have only focused on autosomal SNPs e.g. [9]–[11].

The circular mitochondrial DNA (mtDNA) comprises 16,569 bps. Somatic cells usually harbor about 1,000 to 10,000 mtDNA molecules [12]. mtDNA encodes 37 genes of which 13 are protein coding subunits of the oxidative phosphorylation system (OXPHOS) [13]. In addition, mtDNA consists of a 1,100 bps non-coding control region known as the mitochondrial displacement (D)-loop [13]. The D-loop consists of two to three hypervariable parts [14], [15]. Both transcription and replication are coordinated at the D-loop [12] (Table 1).

**Table 1:** Functionally relevant regions of the D-loop.

Region a	Description/Function	Location Start ^b^	Location End ^b^	Reference	Number of detected variants in location	Mean number of variants per case	Mean number of variants per control	p-value
HV1a	Hypervariable regions	m.16024	m.16365	[14]	126	2.39	2.39	0.977
HV1b		m.16024	m.16382	[45]	127	2.39	2.40	0.953
HV2a		m.73	m.340	[14]	69	4.25	4.33	0.686
HV2b		m.57	m.371	[45]	73	4.30	4.38	0.707
HV3		m.438	m.574	[15]	35	0.75	0.64	0.232
Mt5 (CE)	Intra- and interspecific control element (CE)	m.16194	m.16208	[46]	1	0.01	0	0.319
Mt3 (L-strand CE)	*cis* elements which were first detected in 5' region of nuclear encoded mitochondrial OXPHOS genes, and later on also in the mtDNA D-loop; these elements are potentially involved in a coordinated expression of nuclear-encoded and mtDNA OXPHOS genes	m.16499	m.16506	[47]	0	0	0	NaN
Mt4 (L-strand CE)		m.371	m.379	[47]	1	0	0.01	0.319
Mt3 (H-strand CE)		m.384	m.391	[47]	1	0.02	0	0.083
mtTF1 BS	binding sites (BS) of mitochondrial transcription factor A (TFAM, formerly known as mtTF1)	m.233	m.260	[48]	12	0.12	0.09	0.537
mtTF1 BS		m.276	m.303	[48]	5	0.12	0.12	1.000
mtTF1 BS		m.418	m.445	[48]	0	0	0	NaN
mtTF1 BS		m.525	m.552	[48]	4	0.01	0.02	0.178
LSP (including mtTF1 BS) ^c^	Light strand and heavy strand promoters	m.392	m.445	[49]	1	0.01	0.01	0.563
HSP1		m.545	m.567	[49]	4	0.02	0.01	0.414
HSP1 (including mtTF1 BS)		m.525	m.567	- -	6	0.02	0.02	0.738
HSP2 ^d^		m.632	m.655	[27]	1	0	0.01	0.319
CSB1	potentially involved in direction of transcription termination and heavy strand primer formation	m.210	m.234	[41]	8	0.12	0.06	0.101
CSB2		m.299	m.315	[41], [50]	9	1.48	1.52	0.368
CSB3		m.346	m.363	[41]	1	0.01	0	0.319
ETAS1	potentially involved in premature termination of heavy strand synthesis	m.16081	m.16140	[41]	12	0.39	0.36	0.578
ETAS2		m.16294	m.16356	[41]	25	0.59	0.73	0.103
TAS		m.16157	m.16172	[42], [51]	8	0.12	0.11	0.878

a functionally relevant regions of the D-loop adapted from www.mitomap.org [32], ^b^mtDNA position according to rCRS [29], **^c^**LSP region alone also indicated at m.392 to m.445 [49], ^d^HSP2 was indicated at www.mitomap.org [32] as m.645 [52]–[54], however, more recent investigation mapped start of HSP2 at m.644 [26], [27]; m.632 to m.655 was selected as HSP2 region, as Lodeiro et al. [27] randomized these 24 nucleotides around the transcription start and did not detect transcription *in vitro*, thus, these 24 nucleotides might be important HSP2 control elements (e.g. transcription factor binding sites).

CE, control element; CSB, conserved sequence block; ETAS, extended termination associated sequence, HSP, heavy strand promoter; HV, hypervariable region; LSP, light strand promoter; mtTF1 BS, mitochondrial transcription factor A (TFAM) binding side (TFAM, formerly known as mtTF1), OXPHOS, oxidative phosphorylation; TAS, termination associated sequence.

As mtDNA is exclusively maternally inherited, variation in mtDNA might contribute to the above mentioned higher correlation in BMI between mothers and their offspring. Up to date, only two GWAS of mtDNA variants in association with BMI have been performed [16], [17]. Yang et al. reported association of the mitochondrial haplogroup X with a lower BMI in a sample of 2,286 unrelated adult Caucasians [16]. The finding was not confirmed in an independent sample [16]. A GWAS on both European-American and African-American case-control (CC) samples of obese and lean children did not reveal association of BMI with any mtDNA variant or with heteroplasmy [17].

In the current analysis, we analysed GWAS data of mtDNA variants in extreme early onset obesity by using a CC sample of 1,158 extremely obese children and adolescents and 435 lean adult controls of German descent. Our findings were followed-up in 7,014 German population-based adults. Moreover, in a sub-sample of 384 individuals of the initial CC sample, the D-loop was re-sequenced in order to detect further variants potentially associated with obesity.

## Subjects and Methods

### Study subjects

#### Ethics statement

Written informed consent was given by all participants. The study was approved by the Ethics Committees of the Universities of Marburg, Essen, Greifswald and Kiel, and the Bavarian Medical Association. It was conducted in accordance with the Declaration of Helsinki.

#### Discovery GWAS sample

The CC GWAS sample consisted of 1,158 (extremely) obese children and adolescents and 435 normal weight or lean adult controls. The controls and 453 cases were derived from a CC GWAS sample [18]. The additional 705 cases were index cases from a family-based GWAS sample of 705 obesity trios (one (extremely) obese child or adolescent [ = index case] and both biological parents) [18]. Lean adults as controls who were never overweight or obese during childhood (as assessed by interview) were used as this was thought to reduce the chances of misclassification compared with the use of lean children as controls who might become overweight or obese in adulthood [19]. The measured body mass index (BMI; in kg/m^2^) was assessed for extremeness using age- and sex-specific percentile criteria for the German population from the National Nutrition Survey I [20]. According to this reference population, 84% of all cases were extremely obese (BMI ≥99^th^ percentile). The lean controls had a mean BMI of 18.31±1.11 kg/m^2^ (Table S1).

#### Confirmation GWAS sample

For confirmation of initial findings, three population-based adult GWAS samples were used (n = 7,014, Table S1). (1) KORA: this sample is a sub-sample of KORA F4, which is an epidemiological study group of the region of Augsburg (Cooperative Health Research in the Region of Augsburg) [21] and comprised 1,743 adult participants (890 females). (2) SHIP: “The Study of Health in Pomerania” is a population-based project in Northeast Germany comprising 4,308 individuals aged 20 to 79 years at recruitment. Of these, 4,073 individuals (2,067 females) were genotyped with the Affymetrix Genome-Wide Human SNP Array 6.0 and included in the analysis [22]. (3) POPGEN: the 1,198 individuals (524 females) of POPGEN (age: 19 to 77 years) genotyped with the Affymetrix Array 6.0 are from a population-genetic research project founded at the University Hospital of Schleswig-Holstein [23]. 738 subjects (336 females) were recruited via the local population registry and 460 (188 females) as blood donors. BMI of the individuals recruited via the local population registry was estimated by self-report, while BMI of the blood donors was measured.

As simulations have shown that genetic markers with an effect in the extremes of a trait are detected more solidly within a CC design compared with a linear regression design [24], we converted the population-based samples into a CC sample categorizing all individuals with a BMI ≥30 kg/m^2^ as obese cases (n = 1,697) and those with a BMI <25 kg/m^2^ as normal weight and lean controls (n = 2,373; Table S1).

#### D-loop sample

The D-loop was re-sequenced in 192 extremely obese cases and 192 lean controls. These individuals were derived from the initial CC GWAS sample apart from 14 cases and six controls. Mean age and BMI were similar to those found in the initial CC GWAS sample (Table S1).

### Molecular genetic analysis

#### Genotyping

All individuals were genotyped by the Affymetrix Genome-Wide Human SNP Array 6.0. This array covers 115 to 119 mtDNA SNPs (Table S2). The following quality control (QC) criteria were applied: (1) SNP call-rate above 95%, (2) minor allele frequency (MAF) above 1%, and (3) cluster graphs checked independently by two raters for clear separation of both alleles. Only 32 to 40 SNPs passed these criteria as most of the mtDNA SNPs were monomorphic or had a very low MAF (Table S2).

#### Variant detection by re-sequencing (Sanger) of mitochondrial D-loop

Primers were selected according to Cardoso et al. [25] (Fig. S1). Instead of H616 [25], H715 was chosen to include the sequence of the second heavy strand promoter (HSP2), whose start is located at m.644 [26], [27], i.e. outside of the actual D-loop (m.576 to m.16024, [28]) (Fig. S1). The outer primers L15988 and H715 were used for amplification of the D-loop (primer annealing temperature: 65°C).

Re-sequencing was performed by *LGC Genomics Berlin, Germany* using all four primers. Received electro-pherograms were evaluated manually using *Seqman Pro* (v.10.1.0 (174), 419, DNASTAR, Inc., Madison (WI), USA). The D-loop sequence of the revised Cambridge Reference Sequence (rCRS, [29]) was copied into a *Microsoft Office Excel 2007 (Microsoft Coop., Redmond (WA))* sheet, so that each cell comprised ten nucleotides and each line 60 nucleotides. Deviations from this reference were noted below the reference in a separate *Excel* sheet for each individual. Evaluation of electropherograms was performed by two independent raters. Discrepancies were resolved unambiguously by either reaching consensus or re-sequencing.

#### Variant detection by re-sequencing (Sanger) of complete mitochondrial DNA

Complete mtDNA was re-sequenced in five cases and five controls of the discovery GWAS sample by *Seqlab Göttingen, Germany*. Evaluation of received electropherograms was performed manually as described above.

### Determination of haplogroups

The haplogroup of each individual of the discovery and confirmation sample was determined with HaploGrep [30], [31]. For this analysis, HaploGrep software provided at http://haplogrep.uibk.ac.at/was downloaded. All available mtDNA SNPs from each individual (32 to 40 mtDNA SNPs, Table S2/Table S3) were entered. Each individual's haplogroup was determined based on PhyloTree.org (mtDNA tree build 11, [31]) which is implemented in the software. Only those individuals whose haplogroup quality value was rated ≥90% were included into statistical analyses. At this quality threshold haplogroup assignment is quite reliable according to HaploGrep's manual. For association testing, haplogroups were assigned to major haplogroups.

### Statistic tests

For discovery, association testing was performed using Fisher's two-sided exact test for both single SNP and haplogroup analysis. Analyses were also performed stratified by gender. Odds ratios and confidence intervals were determined. For independent confirmation, nominally associated SNPs or haplogroups were followed-up in the confirmation sample – in the whole sample or stratified by gender depending on the discovery finding.

Frequencies of detected variants by D-loop re-sequencing were compared between cases and controls using Fisher's two-sided exact test. Moreover, average number of variants in 23 defined functionally relevant regions of the D-loop (adapted from www.mitomap.org; Table 1, [32]) was compared between cases and controls with a t-test.

## Results

### Single SNP analysis

Association analysis was performed with 35 mtDNA SNPs in the whole discovery GWAS sample of 1,158 (extremely) obese children and adolescents and 435 lean and normal weight adult controls. Five further SNPs were analyzed in this sample excluding the 705 cases from the family-based trio GWAS sample, as these five SNPs did not pass QC in this sample (Table S3). Nominal association was found for m.8994G/A (p = 0.002), whose minor allele A was more frequent among the controls (3.92% vs. 1.30%; Table 2). m.8994 is located in the *ATP6* gene. The G/A transition at m.8994 is synonymous. Stratified by gender, nominal association was found for both male and female subjects (Table 2). In female subjects, nominal association was found for three further SNPs, while in male subjects one further SNP was nominally associated (Table 2).

**Table 2:** Nominally associated mtDNA SNPs in discovery and follow-up in confirmation.

	Discovery					Confirmation				
SNP a	MAF cases [%] b	MAF controls [%] b	Odds Ratio	Confidence Interval c	p-value d	MAF cases [%] b	MAF controls [%] b	Odds Ratio	Confidence Interval c	p-value d
**All**	**n = 1,158**	**n = 435**				**n = 1,697**	**n = 2,373**			
m.8994G/A	1.30	3.92	0.32	0.15–0.69	**0.002**	3.24	2.41	1.36	0.92–2.02	0.120
**Males**	**n = 508**	**n = 171**				**n = 828**	**n = 930**			
m.8994G/A	0.79	2.94	0.26	0.05–1.23	**0.048**	2.78	2.16	1.30	0.68–2.51	0.441
m.11674C/T	0.59	3.51	0.16	0.03–0.78	**0.010**	2.06	1.63	1.27	0.63–2.56	0.593
**Females**	**n = 650**	**n = 264**				**n = 869**	**n = 1,443**			
m.4769A/G	3.38	0.76	4.60	1.12–40.6	**0.022**	2.99	2.36	1.28	0.76–2.15	0.348
m.8994G/A	1.69	4.55	0.36	0.14–0.91	**0.019**	3.68	2.57	1.45	0.87–2.41	0.132
m.12612A/G	8.00	2.88	0.58	0.36–0.95	**0.023**	10.60	9.01	1.20	0.90–1.59	0.216
m.13708G/A	9.12	13.69	0.61	0.38–0.98	**0.040**	10.87	12.81	1.08	0.83–1.42	0.238

a mtDNA position according to rCRS [29].

b MAF, minor allele frequency.

c 95% confidence of odds ratio for minor allele.

d Fisher's exact test, two-sided, p-values below 0.05 are highlighted in bold.

Follow-up of m.8994G/A in a sample of 1,697 obese cases and 2,373 normal weight controls from three adult population-based GWAS samples did not lead to an independent confirmation of the initial finding. SNPs initially associated in only female or male subjects could neither be confirmed. In addition, direction of effect for most of these SNPs was different between discovery and confirmation (Table 2).

### Haplogroup analysis

Using HaploGrep [30] based on phylotree build 11 [31], we identified 80 haplogroups with a quality value ≥90%. This quality threshold was reached by 96% of all study subjects from both discovery and confirmation. Most of the identified haplogroups (94% to 95% depending on sample) could be assigned to eight European major haplogroups (H, U, T, V, J, K, W, X).

For discovery analysis, we detected nominal association with obesity for haplogroup W (p = 0.034; Table 3, Table S4), which was – just as the minor allele A of m.8994 – more frequent among the lean adult controls. All individuals of haplogroup W were minor allele carriers of m.8994. Stratified by gender, nominal association of haplogroup W remained only for male subjects (p = 0.012). Among the females we detected nominal association for haplogroup J (p = 0.032). Compared to the single SNP GWAS findings, none of the initially associated haplogroups could be confirmed in the independent sample, and directions of effect were opposite (Table 3).

**Table 3:** Nominally associated mt haplogroups in discovery and follow-up in confirmation.

	Discovery					Confirmation				
Haplo-group a	Frequency Cases [%]	Frequency Controls [%]	Odds Ratio	Confidence Interval b	p-value c	Frequency Cases [%]	Frequency Controls [%]	Odds Ratio	Confidence Interval b	p- value c
**all**	**n = 1,114**	**n = 422**				**n = 1,623**	**n = 2,271**			
W	1.17	2.84	0.40	0.17–0.97	**0.034**	2.71	1.94	1.41	0.90–2.20	0.126
**males**	**n = 491**	**n = 163**				**n = 793**	**n = 890**			
W	0.41	3.07	0.13	0.01–0.81	**0.012**	2.14	1.69	1.28	0.60–2.77	0.592
**females**	**n = 623**	**n = 259**				**n = 830**	**n = 1,381**			
J	8.19	13.13	0.58	0.36–0.94	**0.032**	10.96	9.27	1.20	0.89–1.61	0.211

a only individuals with HaploGrep's quality ≥90% were included (∼96% of all individuals).

b 95% confidence interval for odds ratio.

c Fisher's exact test, two-sided, p-values below 0.05 are highlighted in bold.

### Re-sequencing of mtDNA D-loop

We re-sequenced the D-loop in 192 extremely obese cases and 192 lean controls, which were derived from the initial discovery sample apart from 20 individuals. This was done, because prior complete re-sequencing of mtDNA of 10 individuals (eight of these had haplogroup W) revealed that among the individuals of haplogroup W inter-individual variability in the D-loop (Table S5) was larger compared with the coding region (Table S6). Moreover, the coverage of the D-loop by only one SNP on the SNP array (Table S3) was insufficient for association analysis for D-loop variants. Finally, the D-loop is an important control region pertaining to transcription and replication of mtDNA, and variation in this region might have an impact on these processes and interfere with body weight.

We excluded one case and one control each from the 384 individuals whose D-loop was re-sequenced from further analysis, as we detected 9 and 4 clearly visible point heteroplasmies. Point heteroplasmies are usually rather infrequent especially in blood cells [33], [34], from which DNA was extracted, and might be an indication for contamination with foreign DNA [35].

In the remaining 382 individuals we detected a total of 252 deviations from the rCRS [29], four of which were not located in the actual D-loop (i.e. between m.576 and m.16024, Table S7/Table S8). Of these, 223 were single nucleotide exchanges at 213 positions, as at 10 positions tri-allelic exchanges were present, three were complex nucleotide exchanges (i.e. a combination of a single nucleotide exchange and an insertion as for instance m.16183A/CC), 18 were insertions and eight were deletions. A major part of the detected variants have been already described (www.mitomap.org, last edited on Apr 23, 2013, Table S7/Table S8) [32].

**Figure 1:**
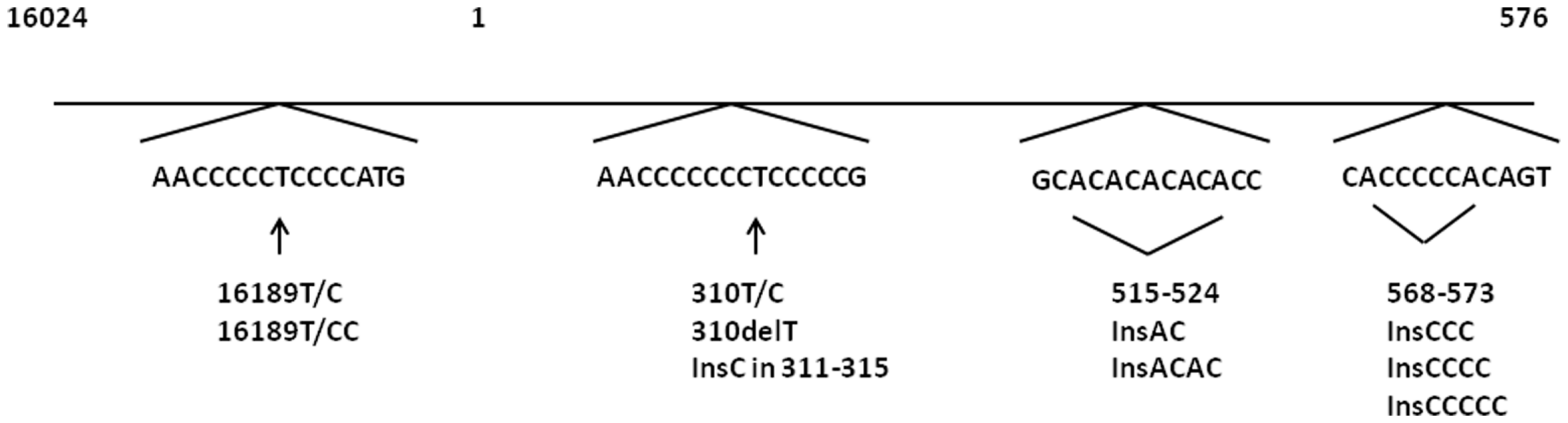
Detected length heteroplasmies. Detection of length heteroplasmies, i.e. mixtures of various lengths of a certain mtDNA region in one individual, occurred at four locations in the D-loop (m.16024 to m.576), predominantly at poly-C tracts. Numbering according to rCRS [29].

Each one point heteroplasmy was detected in four cases and nine controls. Moreover, we detected length heteroplasmies at four previously known length heteroplasmic mtDNA regions (Fig. 1).

The average number of variants per individual was 8.3. This frequency did not differ between cases and controls (p = 0.989). We also compared the frequencies of each of the 252 detected variants between cases and controls, and found m.16292C/T (p = 0.007) and m.16189T/C (p = 0.048) to be nominally associated with obesity. m.16292C/T was present only in eight controls, of which five had haplogroup W, whose frequency was nominally higher in the initial CC GWAS sample and tended to be higher among the controls of the D-loop sample (p = 0.062). By contrast, m.16189T/C had a higher frequency among the cases (17% vs. 9%).

A transition at m.16189 might create an uninterrupted poly-C tract of 10 Cs (Fig. 1), in case no further transition has occurred between m.16184 and m.16193. Among the cases, 15% had such an uninterrupted poly-C tract, while only 10% among the controls (p = 0.116).

We detected no differences when comparing average number of variants per individual between cases and controls for the 23 functionally relevant D-loop locations (Table 1).

## Discussion

Due to its maternal inheritance, we addressed the question if variation in mtDNA might contribute to the observed larger correlation for BMI between mothers and their offspring than between fathers and their offspring [4], [6], [7] or maternal versus paternal half-brothers [8]. Therefore, we performed an association study for mtDNA SNPs predominantly from the mtDNA coding region in extremely obese children and adolescents versus lean adult controls and re-sequenced the D-loop in a sub-sample of this CC study group.

For the coding region variants, no single variant or haplogroup was robustly associated with obesity. The results are in accordance with a previous study [17]. Yang et al. showed association of haplogroup X with lower BMI in a sample of 2,286 unrelated adult Caucasians; however, these data were not confirmed in an independent sample [16]. In our study, haplogroup X was not associated with BMI/leanness in the young or the adult sample. Nevertheless, in the adults the direction of effect was the same as described previously (Table 3; [16]).

We considered that our initial findings were spurious, as the direction of effect of each haplogroup and of all but one SNP differed between discovery and confirmation (Table 2). However, an additional explanation might be that different mtDNA SNPs and/or haplogroups might be relevant for children and adolescents than for adults pertaining to BMI [18]. This could explain why we were not able to replicate association of haplogroup X [16] with low BMI in the discovery (p = 1.0), while a minor trend (p = 0.12, consistence of direction of risk allele) was found in the adults (confirmation). Obviously, based on the sample sizes we cannot exclude the existence of associations of small effect sizes.

Haplogroup assignment was performed with HaploGrep [30] based on Phylotree built 11 [31] with the genotype information of the up to 40 mtDNA SNPs from the SNP array. Bandelt et al. rated HaploGrep to be more sophisticated than the other programs which are able to assign haplogroups automatically [36]. All major European haplogroups – except haplogroup I – were found, and their frequencies were in accordance with those expected among West Europeans (www.mitomap.org, [32]) or Germans ([37]; Table S9). Individuals could not be assigned to haplogroup I, as SNPs at m.10034, m.16129 and m.16391 leading to haplogroup I or any variant which would have led to a sub-haplogroup of haplogroup I were absent from the SNP array (www.phylotree.org, built 11; Fig. S2; [31]). Haplogroup I branches-off of N1e'l (www.phylotree.org, built 11; Fig. S2; [31]). In West Europeans and Germans, Haplogroup N occurs with a frequency of 1% (www.mitomap.org, [32]) and 0.6% [37], respectively, but among the individuals of the current study samples, the frequency was 2.5 to 3.5% (Table S9). Thus, individuals actually belonging to haplogroup I might have “remained” in haplogroup N1 (Fig. S2).

Haplogroup association testing was restrained to major haplogroups, because of the limited number of SNPs on the SNP array which disabled a refined haplogroup determination for some individuals. Moreover, given a major haplogroup is present at a low percentage in a population as for instance haplogroup W in the present study samples (∼2%, Table S9), refined haplogroup association testing would have to be done in a sample with a much larger sample size as those of the present study samples. Nevertheless, variants biologically relevant for obesity might be found in the sub-haplogroups and hence these variants or sub-haplogroups might have been masked by association testing of only the respective major haplogroup.

Pertaining to the D-loop variants, two (m.16292C/T and m.16189T/C) of the 252 detected variants were nominally associated with obesity among the 191 cases and 191 controls of the D-loop sample. m.16292C/T was only found in eight controls of which five had haplogroup W that was initially overrepresented among the controls of the CC GWAS sample (p = 0.048; D-loop sample: p = 0.062). As haplogroup W could not be confirmed independently in the present study, a follow-up of this variant does not seem useful.

m.16189T/C is located with a poly-C tract between m.16184 and m.16193. This variant as well as m.16189T/CC and m.16189delT led to an uninterrupted poly-C tract given no other transition or insertion except of C has occurred between m.16184 and m.16193 (Fig. 1). In the present study, frequency of the uninterrupted poly-C tract tended to be higher among the cases. Parker et al., by contrast, reported the uninterrupted poly-C tract to be associated with leanness among 161 Australian mothers and their 20-year-old offspring [38].

Moreover, all individuals of the present D-loop sample with an uninterrupted poly-C tract showed length heteroplasmy at this tract. This might be the result of strand slippage during the replication process generating tracts of variable length of predominantly 10 to 12 Cs [39], [40]. Moreover, length patterns were different between maternal lineages but nearly identical within a maternal lineage [39]. In addition, the termination associated element (TAS, m.16157 to m.16172, Table 1), which is involved in premature determination of the H-strand synthesis in order to create the triple stranded D-loop [28], [41], [42] is located near this C tract. As binding of proteins at the TAS element was shown, binding capacity and thus mtDNA transcription and replication might be influenced by the nearby uninterrupted C tract [43]. Among 837 healthy adult Taiwanese (mean BMI = 24.5 kg/m^2^), the lowest mtDNA content was found among individuals with an uninterrupted C tract compared with individuals of the wild-type or an otherwise interrupted C tracts [44]. Nevertheless, mean BMI between the three groups was similar [44]. Further investigation is needed whether 1) the detected overrepresentation by trend of the uninterrupted C tract in the obese can be confirmed in an independent sample and 2) the uninterrupted C-tract has an influence on mtDNA levels and BMI among Europeans.

Finally, as some D-loop variants only occurred at very low frequencies (in only one or two individuals), a refined association analysis of these variants potentially in combination with the 23 functionally relevant regions in a larger study sample could be subject of further investigation.

All in all, though follow-up of some D-loop variants still is conceivable, our hypothesis of a contribution of variation in the exclusively maternally inherited mtDNA to the observed greater correlations in BMI between mothers and their offspring than between fathers and their offspring could not be substantiated by the findings of the present study.

## Supporting Information

Figure S1 Selection of primers for the re-sequencing of the mtDNA D-Loop.
(DOCX)

Figure S2 Haplogroup I branching off of N1e'l.
(DOCX)

Table S1 Phenotypical characteristics of subjects.
(DOCX)

Table S2 Quality control of SNPs.
(DOCX)

Table S3 SNPs of mtDNA in association with obesity in discovery.
(DOCX)

Table S4 Frequency of major haplogroups in cases and controls in discovery and confirmation.
(DOCX)

Table S5 D-loop variants detected by re-sequencing (Sanger) of complete mtDNA of each five lean and obese individuals.
(DOCX)

Table S6 Coding region variants detected by re-sequencing (Sanger) of complete mtDNA of each five lean and obese individuals.
(DOCX)

Table S7 D-loop variants (single nucleotide exchanges) detected by re-sequencing (Sanger) of mtDNA and frequencies in cases and controls.
(DOCX)

Table S8 D-loop variants (complex nucleotide exchanges, insertions and deletions) detected by re-sequencing (Sanger) of mtDNA and frequencies in cases and controls.
(DOCX)

Table S9 Distribution of haplogroup frequencies (in%) in study samples compared with West Europeans and Germans.
(DOCX)
