# Supplementary material for: Mitochondrial DNA Variants in Obesity
Source: PLoS One. 2014 May 2;9(5):e94882. doi: 10.1371/journal.pone.0094882 (PMC4008486; doi:10.1371/journal.pone.0094882)
Supplement: Figure S1 — Selection of primers for the re-sequencing of the mtDNA D-Loop. (DOCX) [file pone.0094882.s001.docx]

**Figure S1 Selection of primers for the re‐sequencing of the mtDNA D‐Loop**

L15988, 5‘‐AAGTCTTTAACTCCACCATTAGC‐3’; L12, 5‘‐ACATCACGATGGATCACAGGTC‐3‘, H285, 5‘‐GGGGTTTGGTGGAAATTTTTTG‐3‘ selected according to Cardoso et al. (2012); H715, 5‘‐TGGAACGGGGATGCTTGCAT‐3‘ chosen using primer3 (v.0.4.0; http://frodo.wi.mit.edu/) for inclusion of the second heavy strand primer (HSP2) starting position at m.644A (Zollo et al. 2012, Lodeiro et al. 2012), i.e. outside of the actual D-loop (m.576 to m.16024, Anderson et al. 1981).

References:

Anderson S, Bankier AT, Barrell BG, de Bruijn MH, Coulson AR, et al. (1981) Sequence and organization of the human mitochondrial genome. Nature 290(5806):457-65.

Cardoso S, Villanueva-Millán MJ, Valverde L, Odriozola A, Aznar JM, et al. (2012) Mitochondrial DNA control region variation in an autochthonous Basque population sample from the Basque Country. Forensic Sci Int Genet 6(4):e106-8.

Lodeiro MF, Uchida A, Bestwick M, Moustafa IM, Arnold JJ, et al. (2012) Transcription from the second heavy-strand promoter of human mtDNA is repressed by transcription factor A in vitro. Proc Natl Acad Sci U S A 109(17):6513-8.

Zollo O, Tiranti V, Sondheimer N (2012) Transcriptional requirements of the distal heavy-strand promoter of mtDNA. Proc Natl Acad Sci U S A 109(17):6508-12.
