# Supplementary material for: Mitochondrial DNA Variants in Obesity
Source: PLoS One. 2014 May 2;9(5):e94882. doi: 10.1371/journal.pone.0094882 (PMC4008486; doi:10.1371/journal.pone.0094882)
Supplement: Figure S2 — Haplogroup I branching off of N1e'l. (DOCX) [file pone.0094882.s002.docx]

**Figure S2 Haplogroup I branching off of N1e’l**

Screen shot from Phylotree built 11 (van Oven and Kayser 2009) demonstrates haplogroup I as a side branch of haplogroup N1e’l. Nucleotide positions (blue, control region; black, coding region) are relative to the rCRS (Andrews et al. 1999). If not otherwise indicated by the respective nucleotide position, variants are transitions. Italic nucleotide positions are preliminary and likely to be refined once additional sequences become available.

References:

Andrews RM, Kubacka I, Chinnery PF, Lightowlers RN, Turnbull DM, et al. (1999) Reanalysis and revision of the Cambridge reference sequence for human mitochondrial DNA. Nat Genet 23(2):147.

van Oven M, Kayser M (2009) Updated comprehensive phylogenetic tree of global human mitochondrial DNA variation. Hum Mutat 30(2):E386-E394. http://www.phylotree.org.
