# Supplementary material for: Mitochondrial DNA Variants in Obesity
Source: PLoS One. 2014 May 2;9(5):e94882. doi: 10.1371/journal.pone.0094882 (PMC4008486; doi:10.1371/journal.pone.0094882)
Supplement: Table S1 — Phenotypical characteristics of subjects. (DOCX) [file pone.0094882.s003.docx]

**Table S1** **Phenotypical characteristics of subjects**

| **Sample** | **Description** | **Status** | **n total** | **Age [years]** | **BMI [kg/m^2^]** | **BMI SDS ^a^** |
| --- | --- | --- | --- | --- | --- | --- |
|  |  |  | {% female} | {female} | {female} | {female} |
|  |  |  |  | (male) | (male) | (male) |
|  | | |  | **mean ± SD** | **mean ± SD** | **mean ± SD** |
| Discovery Case-control (CC) GWAS sample ^b^ | (extremely) obese children and adolescents | cases | 1,158 | 13.79 ± 3.35 | 32.45 ± 6.19 | 4.35 ± 2.04 |
|  |  |  | {55.81} | {13.91 ± 3.34} | {32.66 ± 6.39} | {4.55 ± 2.10} |
|  |  |  |  | (13.65 ± 3.36) | (32.18 ± 5.93) | (4.10 ± 1.95) |
|  | Lean or normal weight subjects | controls | 435 | 26.08 ± 5.75 | 18.31 ± 1.11 | -1.45 ± 0.34 |
|  |  |  | {61.00} | {26.54 ± 6.37} | {17.58 ± 0.95} | {-1.35 ± 0.30} |
|  |  |  |  | (25.38 ± 4.57) | (18.86 ± 0.94) | (-1.60 ± 0.36) |
| Confirmation CC GWAS sample ^c^ | obese subjects (BMI ≥ 30) | cases | 1,697 | 55.08 ± 12.87 | 33.57 ± 3.47 | 2.42 ± 1.10 |
|  |  |  | {51.21} | {55.27 ± 12.89} | {34.13 ± 3.67} | {2.49 ± 1.06} |
|  |  |  |  | (54.87 ± 12.86) | (32.98 ± 3.15) | (2.36 ± 1.14) |
|  | normal weight subjects  (BMI < 25) | controls | 2,373 | 46.15 ± 15.37 | 22.57 ± 1.69 | -0.53 ± 0.55 |
|  |  |  | {60.81} | {45.23 ± 14.71} | {22.27 ± 1.75} | {-0.42 ± 0.52} |
|  |  |  |  | (47.57 ± 16.26) | (23.03 ± 1.47) | (-0.71 ± 0.54) |
| D-loop  Sample ^d^ | (extremely) obese children and adolescents | cases | 192 | 13.87 ± 3.05 | 33.00 ± 7.24 | 4.49 ± 2.38 |
|  |  |  | {52.60} | {13.79 ± 3.11} | {33.37 ± 8.20} | {4.75 ± 2.67} |
|  |  |  |  | (13.95 ± 2.99) | (32.59 ± 6.01) | (4.19 ± 1.98) |
|  | Lean or normal weight subjects | controls | 192 | 25.50 ± 3.95 | 18.37 ± 1.09 | -1.46 ± 0.33 |
|  |  |  | {45.31} | {24.93 ± 3.82} | {17.61 ± 0.71} | {-1.31 ± 0.24} |
|  |  |  |  | (25.97 ± 4.01) | (19.01 ± 0.93) | (-1.59 ± 0.34) |

^a^ BMI SDS calculation based on reference data of the German National Nutrition Survey I (Hebebrand et al. 1994)

^b^ subjects are derived from a CC GWAS sample comprising 453 (extremely) obese children and adolescents and 435 lean adult controls (Scherag et al. 2010) and a family-based GWAS sample of 705 trios (i.e. one (extremely) obese child or adolescent as index patient and both biological parents; Scherag et al. 2010); only the index patient was included in the present sample

^c^ Subjects are derived from three German population-based study cohorts (KORA, Rückert et al. 2011; SHIP, Völzke et al. 2011; POPGEN, Nöthlings and Krawczak 2012) comprising in total 7,014 individuals

^d^ All individuals apart from 14 cases and six controls are from the discovery CC GWAS sample; analyses of Affymetrix Genome-Wide Human SNP Array 6.0 failed for these 20 individuals.

References:

Hebebrand J, Heseker H, Himmelmann GW, Schäfer H, Remschmidt H (1994) Altersperzentilen für den Body Mass Index aus Daten der Nationalen Verzehrstudie einschließlich einer Übersicht zu relevanten Einflussfaktoren. Aktuelle Ernährungsmedizin 19: 259-265.

Nöthlings U, Krawczak M (2012) [PopGen. A population-based biobank with prospective follow-up of a control group]. Bundesgesundheitsblatt Gesundheitsforschung Gesundheitsschutz 55(6-7):831-5. German.

Rückert IM, Heier M, Rathmann W, Baumeister SE, Döring A, et al. (2011) Association between markers of fatty liver disease and impaired glucose regulation in men and women from the general population: the KORA-F4-study. PLoS One. 6(8):e22932.

Scherag A, Dina C, Hinney A, Vatin V, Scherag S, et al. (2010) Two new Loci for body-weight regulation identified in a joint analysis of genome-wide association studies for early-onset extreme obesity in French and german study groups. PLoS Genet 22;6(4):e1000916.

Völzke H, Alte D, Schmidt CO, Radke D, Lorbeer R, et al. (2011) Cohort profile: the study of health in Pomerania. Int J Epidemiol. 40(2):294-307.
