# Supplementary material for: Mitochondrial DNA Variants in Obesity
Source: PLoS One. 2014 May 2;9(5):e94882. doi: 10.1371/journal.pone.0094882 (PMC4008486; doi:10.1371/journal.pone.0094882)
Supplement: Table S2 — Quality control of SNPs. (DOCX) [file pone.0094882.s004.docx]

**Table S2 Quality control of SNPs**

| **Sample** | **CC^a^** | **TRIOS^b^** | **KORA^c^** | **SHIP^c^** | **POPGEN^c^** |
| --- | --- | --- | --- | --- | --- |
|  | **n=888** | **n=2,115**  **705 trios** | **n=1,743** | **n=4,073** | **n=1,198** |
|  |  |  |  |  |  |
| number of mtDNA SNPs genotyped ^a^ | 119 | 115 | 115 | 115 | 115 |
| **1^st^ SNP-QC criterion** | **sample call-rate per SNP ≥ 95 %** | | | | |
| number of SNPs failing 1^st^ SNP-QC criterion | 3 | 6 | 6 | 2 | 11 |
| % of SNPs failing 1^st^ SNP-QC criterion | 2.52 | 5.22 | 5.22 | 1.74 | 9.57 |
| **2^nd^ SNP-QC criterion** | **MAF ≥ 1 %** | | | | |
|  | in sample | in sample | in sample | in sample | in sample |
| number of SNPs failing 2^nd^ SNP-QC criterion | 76 | 68 | 72 | 71 | 71 |
| % of SNPs failing 2^nd^ SNP-QC criterion | 63.9 | 59.1 | 62.6 | 61.7 | 61.7 |
| **3^rd^ SNP-QC criterion** | **Clear separation of the 2 alleles in the cluster graph of each SNP** | | | | |
| number of SNPs failing 3^rd^ SNP-QC criterion | 1 | 7 | 7 | 17 | 7 |
| % of SNPs failing 3^rd^ SNP-QC criterion | 0.84 | 6.09 | 6.09 | 14.8 | 6.09 |
| number of SNPs left after SNP-QC ^b^ | 40 | 35 | 37 | 32 | 35 |
| % of SNPs left after SNP-QC | 33.6 | 30.4 | 32.2 | 27.8 | 30.4 |

^a^ case-control GWAS sample comprising 453 (extremely) obese children and adolescents and 435 lean adult controls (Scherag et al. 2010)

^b^ each trio consists of one (extremely) obese child or adolescent as index patient and both biological parents (Scherag et al. 2010)

^c^ population-based samples (KORA, Cooperative Health Research in the Region of Augsburg, Rückert et al. 2011; SHIP, The Study of Health in Pomerania, Völzke et al. 2011; POPGEN, population-genetic research project founded at the University Clinic of Schleswig-Holstein; Nöthlings and Krawczak 2012)

References:

Nöthlings U, Krawczak M (2012) [PopGen. A population-based biobank with prospective follow-up of a control group]. Bundesgesundheitsblatt Gesundheitsforschung Gesundheitsschutz 55(6-7):831-5. German.

Rückert IM, Heier M, Rathmann W, Baumeister SE, Döring A, et al. (2011) Association between markers of fatty liver disease and impaired glucose regulation in men and women from the general population: the KORA-F4-study. PLoS One. 6(8):e22932.

Scherag A, Dina C, Hinney A, Vatin V, Scherag S, et al. (2010) Two new Loci for body-weight regulation identified in a joint analysis of genome-wide association studies for early-onset extreme obesity in French and german study groups. PLoS Genet 22;6(4):e1000916.

Völzke H, Alte D, Schmidt CO, Radke D, Lorbeer R, et al. (2011) Cohort profile: the study of health in Pomerania. Int J Epidemiol. 40(2):294-307.
