# Supplementary material for: Mitochondrial DNA Variants in Obesity
Source: PLoS One. 2014 May 2;9(5):e94882. doi: 10.1371/journal.pone.0094882 (PMC4008486; doi:10.1371/journal.pone.0094882)
Supplement: Table S3 — SNPs of mtDNA in association with obesity in discovery. (DOCX) [file pone.0094882.s005.docx]

Table S3 SNPs of mtDNA in association with obesity in discovery

| **Position on**  **mtDNA according to rCRS** | **rs number** | **Minor**  **allele ^a^** | **Major**  **allele ^a^** | **MAF cases**  **[%] ^b^** | **MAF**  **controls**  **[%] ^b^** | **Odds**  **ratio**  **for minor allele** | **Confidence**  **Interval** | **p-value ^c^** |
| --- | --- | --- | --- | --- | --- | --- | --- | --- |
|  |  |  |  | **n=1,158** | **n=435** |  |  |  |
| m.1438 | rs2001030 | **A** | G | 2.94 | 2.76 | 1.06 | 0.53-2.28 | 1.000 |
| m.1700 | rs2854126 **^d^** | C | **T** | 1.12 | 1.62 | 0.69 | 0.17-2.54 | 0.573 |
| m.1811 | rs28358576 | G | **A** | 13.04 | 13.56 | 0.96 | 0.69-1.35 | 0.803 |
| m.1888 | rs28358577 **^d^** | A | **G** | 11.04 | 8.28 | 1.37 | 0.86-2.22 | 0.175 |
| m.2706 | rs2854128 | **A** | G | 46.28 | 45.73 | 1.02 | 0.81-1.28 | 0.865 |
| m.3010 | rs3928306 | A | **G** | 24.37 | 26.68 | 0.89 | 0.68-1.15 | 0.362 |
| m.3197 | rs2854131 | C | **T** | 8.23 | 7.64 | 1.08 | 0.71-1.69 | 0.757 |
| m.4580 | rs28357975 | A | **G** | 3.46 | 3.70 | 0.93 | 0.50-1.81 | 0.879 |
| m.4769 | rs3021086 | **A** | G | 3.11 | 1.84 | 1.71 | 0.77-4.30 | 0.229 |
| m.7028 | rs2015062 | **C** | T | 45.76 | 44.76 | 1.04 | 0.83-1.31 | 0.734 |
| m.8994 | rs28358887 | A | **G** | 1.30 | 3.92 | 0.32 | 0.15-0.69 | **0.002** |
| m.9055 | rs28358268 **^d^** | A | **G** | 6.62 | 7.60 | 0.86 | 0.50-1.49 | 0.603 |
| m.9123 | rs28358270 | A | **G** | 1.56 | 1.61 | 0.96 | 0.38-2.75 | 1.000 |
| m.9698 | rs9743 | C | **T** | 6.65 | 8.35 | 0.78 | 0.51-1.22 | 0.272 |
| m.10238 | rs28358275 | C | **T** | 3.11 | 2.76 | 1.13 | 0.57-2.41 | 0.869 |
| m.10463 | rs28358279 | C | **T** | 10.45 | 8.99 | 1.18 | 0.80-1.77 | 0.454 |
| m.10550 | rs28358280 | G | **A** | 6.13 | 7.16 | 0.85 | 0.54-1.36 | 0.490 |
| m.11251 | rs3915952 | G | **A** | 19.43 | 21.43 | 0.88 | 0.67-1.17 | 0.398 |
| m.11299 | rs28358285 | C | **T** | 6.07 | 6.93 | 0.87 | 0.55-1.40 | 0.562 |
| m.11467 | rs2853493 | G | **A** | 21.56 | 21.43 | 1.01 | 0.77-1.33 | 1.000 |
| m.11674 | rs28358286 | T | **C** | 1.39 | 2.99 | 0.46 | 0.20-1.04 | 0.055 |
| m.11719 | rs2853495 | A | **G** | 49.70 | 50.34 | 0.97 | 0.78-1.22 | 0.822 |
| m.11812 | rs3088053 **^d^** | G | **A** | 6.74 | 6.53 | 1.04 | 0.59-1.83 | 1.000 |
| m.11914 | rs2853496 | A | **G** | 2.34 | 1.15 | 2.06 | 0.77-6.89 | 0.162 |
| m.12007 | rs2853497 | A | **G** | 1.64 | 2.07 | 0.79 | 0.34-1.99 | 0.527 |
| m.12308 | rs2853498 | G | **A** | 21.78 | 21.61 | 1.01 | 0.77-1.34 | 1.000 |
| m.12612 | rs28359172 | G | **A** | 8.89 | 11.72 | 0.74 | 0.51-1.07 | 0.105 |
| m.12705 | rs2854122 | T | **C** | 7.81 | 7.14 | 1.10 | 0.71-1.74 | 0.750 |
| m.13368 | rs3899498 | A | **G** | 10.11 | 8.51 | 1.21 | 0.81-1.84 | 0.392 |
| m.13617 | rs2853503 | C | **T** | 8.12 | 7.37 | 1.11 | 0.72-1.74 | 0.677 |
| m.13708 | rs28359178 | A | **G** | 10.13 | 12.47 | 0.79 | 0.56-1.14 | 0.203 |
| m.14470 | rs3135030 | C | **T** | 2.60 | 2.07 | 1.26 | 0.58-3.04 | 0.716 |
| m.14905 | rs28357682 | A | **G** | 10.19 | 8.28 | 1.26 | 0.84-1.91 | 0.295 |
| m.15043 | rs28357684 | A | **G** | 4.24 | 2.99 | 1.44 | 0.76-2.91 | 0.309 |
| m.15218 | rs2853506 | G | **A** | 4.40 | 3.22 | 1.39 | 0.75-2.74 | 0.322 |
| m.15326 | rs2853508 | **A** | G | 1.47 | 0.69 | 2.14 | 0.62-11.5 | 0.312 |
| m.15452 | rs3088309 | A | **C** | 18.70 | 19.82 | 0.93 | 0.70-1.25 | 0.616 |
| m.15607 | rs28357372 | G | **A** | 10.19 | 8.51 | 1.22 | 0.82-1.85 | 0.343 |
| m.15924 | rs2853510 | G | **A** | 4.23 | 3.69 | 1.15 | 0.64-2.20 | 0.672 |
| m.16140 | rs3134562 **^d^** | C | **T** | 6.25 | 6.96 | 0.89 | 0.50-1.57 | 0.686 |

^a^ Reference allele of rCRS (Andrews et al. 1999) marked in bold, ^b^ MAF, minor allele frequency, ^c^ Fisher’s exact test, two-sided, p-values below 0.05 are highlighted in bold, ^d^ SNPs that did not pass quality control in family-based trios

Reference:

Andrews RM, Kubacka I, Chinnery PF, Lightowlers RN, Turnbull DM, et al. (1999) Reanalysis and revision of the Cambridge reference sequence for human mitochondrial DNA. Nat Genet 23(2):147.
