# Supplementary material for: Mitochondrial DNA Variants in Obesity
Source: PLoS One. 2014 May 2;9(5):e94882. doi: 10.1371/journal.pone.0094882 (PMC4008486; doi:10.1371/journal.pone.0094882)
Supplement: Table S4 — Frequency of major haplogroups in cases and controls in discovery and confirmation. (DOCX) [file pone.0094882.s006.docx]

Table S4 Frequency of major haplogroups in cases and controls in discovery and confirmation

|  | **Discovery** | | | | | **Confirmation** | | | | | |
| --- | --- | --- | --- | --- | --- | --- | --- | --- | --- | --- | --- |
| **Haplo-**  **group ^a, b^** | **Frequency**  **Cases**  **[%]** | **Frequency**  **Controls**  **[%]** | **Odds**  **Ratio** | **Confidence Interval ^c^** | **p-value ^d^** | **Frequency**  **Cases**  **[%]** | **Frequency**  **Controls**  **[%]** | **Odds**  **Ratio** | **Confidence Interval ^c^** | | **p-value ^d^** |
|  | **n=1,114** | **n=422** |  |  |  | **n=1,623** | **n=2,271** |  |  |  |  |
| A | 0 | 0 | - | - | - | 0.12 | 0.13 | 0.93 | 0.08-8.15 | | 1.000 |
| B | 0.00 | 0.24 | 0.00 | 0.00-14.65 | 0.275 | 0.62 | 0.48 | 1.27 | 0.48-3.31 | | 0.659 |
| D | 0 | 0 | - | - | - | 0.12 | 0.04 | 2.80 | 0.15-165 | | 0.575 |
| H | 46.23 | 45.73 | 1.00 | 0.80-1.26 | 0.909 | 42.21 | 43.06 | 0.97 | 0.85-1.10 | | 0.599 |
| J | 9.16 | 12.09 | 0.73 | 0.50-1.06 | 0.104 | 10.41 | 9.91 | 1.06 | 0.85-1.31 | | 0.628 |
| K | 6.37 | 7.35 | 0.85 | 0.54-1.36 | 0.492 | 3.51 | 3.65 | 0.96 | 0.67-1.37 | | 0.862 |
| L | 0.09 | 0 | Inf | 0.01-Inf | 1.000 | 0.06 | 0.09 | 0.70 | 0.01-13.4 | | 1.000 |
| M | 1.08 | 0.24 | 4.54 | 0.67-195 | 0.129 | 0.37 | 0.66 | 0.56 | 0.18-1.53 | | 0.271 |
| N | 3.32 | 2.61 | 1.27 | 0.63-2.79 | 0.516 | 3.94 | 3.04 | 1.31 | 0.91-1.88 | | 0.129 |
| P | 0.09 | 0.00 | Inf | 0.01-Inf | 1.000 | 0.00 | 0.04 | 0.00 | 0.00-54.5 | | 1.000 |
| R | 0.36 | 0.24 | 1.50 | 0.15-74.2 | 1.000 | 0.37 | 0.44 | 0.84 | 0.25-2.55 | | 0.804 |
| S | 0 | 0 | - | - | - | 0.06 | 0.00 | Inf | 0.04-Inf | | 0.417 |
| T | 10.41 | 8.53 | 1.23 | 0.83-1.88 | 0.293 | 9.74 | 11.14 | 0.86 | 0.69-1.07 | | 0.169 |
| U | 16.16 | 14.93 | 1.09 | 0.79-1.51 | 0.584 | 21.32 | 20.70 | 1.04 | 0.88-1.21 | | 0.661 |
| V | 3.32 | 3.79 | 0.86 | 0.46-1.68 | 0.641 | 3.64 | 3.26 | 1.12 | 0.78-1.61 | | 0.532 |
| W | 1.17 | 2.84 | 0.40 | 0.17-0.97 | **0.034** | 2.71 | 1.94 | 1.41 | 0.90-2.20 | | 0.126 |
| X | 2.24 | 1.42 | 1.58 | 0.63-4.73 | 0.416 | 0.80 | 1.37 | 0.58 | 0.28-1.15 | | 0.124 |
| Z | 0 | 0 | - | - | - | 0.00 | 0.04 | 0.00 | 0.00-54.5 | | 1.000 |

^a^ Haplogroup determined using Affymetrix Genome-Wide Human SNP Array 6.0 data and HaploGrep (Kloss-Brandstätter et al. 2011) based on Phylotree built 11 (van Oven and Kayser 2009), ^b^ only individuals with HaploGrep's quality ≥ 90% were included (~96% of all individuals), ^c^ 95 % confidence interval for odds ratio, ^d^ Fisher's exact test, two-sided, p-values below 0.05 are highlighted in bold

References:

Kloss-Brandstätter A, Pacher D, Schönherr S, Weissensteiner H, et al. (2011) HaploGrep: a fast and reliable algorithm for automatic classification of mitochondrial DNA haplogroups. Hum Mutat 32(1):25-32.

van Oven M, Kayser M (2009) Updated comprehensive phylogenetic tree of global human mitochondrial DNA variation. Hum Mutat 30(2):E386-E394. http://www.phylotree.org.
