# Supplementary material for: Mitochondrial DNA Variants in Obesity
Source: PLoS One. 2014 May 2;9(5):e94882. doi: 10.1371/journal.pone.0094882 (PMC4008486; doi:10.1371/journal.pone.0094882)
Supplement: Table S5 — D-loop variants detected by re-sequencing (Sanger) of complete mtDNA of each five lean and obese individuals. (DOCX) [file pone.0094882.s007.docx]

Table S5 D-loop variants detected by re-sequencing (Sanger) of complete mtDNA of each five lean and obese individuals

|  |  |  | **Individual ^a^ (Haplogroup ^b^)** | | | | | | | | | |
| --- | --- | --- | --- | --- | --- | --- | --- | --- | --- | --- | --- | --- |
| **Position ^c^** | **Reference**  **Allele ^d^** | **Variant**  **Allele** | **1 (W)** | **2 (W)** | **3 (W)** | **4 (H)** | **5 (HV)** | **6 (W)** | **7 (W)** | **8 (W)** | **9 (W)** | **10 (W)** |
| m.16093 | T | C |  |  |  | C |  |  |  |  |  |  |
| m.16104 | C | A | A |  |  |  |  |  |  |  |  |  |
| m.16140 | T | C |  |  |  |  |  |  |  |  |  |  |
| m.16192 | C | T |  |  | T |  |  |  |  |  |  |  |
| m.16213 | G | A |  |  |  |  |  | A |  |  |  |  |
| m.16221 | C | T |  |  |  | T |  |  |  |  |  |  |
| m.16223 | C | T | T | T | T |  |  | T | T | T | T | T |
| m.16261 | C | T |  |  |  |  |  |  |  |  | T |  |
| m.16286 | C | T |  |  |  |  |  |  |  |  |  | T |
| m.16292 | C | T | T | T | T |  |  |  | T | T | T |  |
| m.16295 | C | T |  |  |  |  |  |  |  | T |  |  |
| m.16301 | C | T |  |  |  |  |  | T |  |  |  |  |
| m.16311 | T | C | C |  |  |  | C |  |  |  |  |  |
| m.16324 | T | C |  |  |  |  |  |  |  |  | C |  |
| m.16325 | T | C |  |  | C |  |  |  |  |  |  |  |
| m.16362 | T | C |  | C |  |  |  |  | C |  |  |  |
| m.16519 | T | C | C | C | C | C |  | C | C | C | C | C |
| m.73 | A | G | G | G | G |  |  | G | G | G | G | G |
| m.94 | G | A |  |  |  |  |  | A |  |  |  |  |
| m.119 | T | C |  |  |  |  |  |  |  | C | C |  |
| m.143 | G | A |  |  |  |  |  |  |  |  |  | A |
| m.152 | T | C |  |  |  |  | C |  |  |  |  |  |
| m.189 | A | G | G | G | G |  |  |  | G | G | G | G |
| m.192 | T | C |  |  |  |  |  |  |  |  |  | C |
| m.194 | C | T | T | T | T |  |  |  | T |  |  | T |
| m.195 | T | C | C | C | C |  |  |  | C | C | C | C |
| m.196 | T | C |  |  |  |  |  |  |  |  |  | C |
| m.199 | T | C | C |  |  |  |  |  |  |  |  |  |
| m.204 | T | C | C | C | C |  |  | C | C | C | C | C |
| m.207 | G | A | A | A | A |  |  | A | A | A | A | A |
| m.263 | A | G | G | G | G | G | G | G | G | G | G | G |
| m.309 |  |  |  |  |  | 309.1C |  |  | 309.1C |  | 309.1C |  |
| m.309 |  |  |  |  |  |  | 309.1CC |  |  |  |  |  |
| m.315 |  |  | 315.1C | 315.1C | 315.1C | 315.1C | 315.1C | 315.1C | 315.1C | 315.1C | 315.1C | 315.1C |

^a^ Individual 1-5 extremely obese children and adolescents; individual 6-10 lean adults

^b^ Haplogroup determined using Affymetrix Genome-Wide Human SNP Array 6.0 data and HaploGrep (Kloss-Brandstätter et al. 2011) based on Phylotree built 11 (van Oven and Kayser 2009); only individuals with HaploGrep's Quality ≥ 90 % were included

^c^ grey shaded positions represent the 40 SNPs present on the Affymetrix Genome-Wide Human SNP Array 6.0

^d^ according to rCRS (Andrews et al. 1999); only deviations from the reference are shown

References:

Andrews RM, Kubacka I, Chinnery PF, Lightowlers RN, Turnbull DM, et al. (1999) Reanalysis and revision of the Cambridge reference sequence for human mitochondrial DNA. Nat Genet 23(2):147.

Kloss-Brandstätter A, Pacher D, Schönherr S, Weissensteiner H, et al. (2011) HaploGrep: a fast and reliable algorithm for automatic classification of mitochondrial DNA haplogroups. Hum Mutat 32(1):25-32.

van Oven M, Kayser M (2009) Updated comprehensive phylogenetic tree of global human mitochondrial DNA variation. Hum Mutat 30(2):E386-E394. http://www.phylotree.org.
