# Supplementary material for: Mitochondrial DNA Variants in Obesity
Source: PLoS One. 2014 May 2;9(5):e94882. doi: 10.1371/journal.pone.0094882 (PMC4008486; doi:10.1371/journal.pone.0094882)
Supplement: Table S6 — Coding region variants detected by re-sequencing (Sanger) of complete mtDNA of each five lean and obese individuals. (DOCX) [file pone.0094882.s008.docx]

**Table S6 Coding region variants detected by re-sequencing (Sanger) of complete mtDNA of each five lean and obese individuals**

|  |  |  | **Individual ^a^ (Haplogroup ^b^)** | | | | | | | | | |
| --- | --- | --- | --- | --- | --- | --- | --- | --- | --- | --- | --- | --- |
| **Position ^c^** | **Reference Allele ^d^** | **Variant Allele** | **1 (W)** | **2 (W)** | **3 (W)** | **4 (H)** | **5 (HV)** | **6 (W)** | **7 (W)** | **8 (W)** | **9 (W)** | **10 (W)** |
| m.709 | G | A | A | A | A |  |  | A | A | A | A | A |
| m.750 | A | G | G | G | G | G | G | G | G | G | G | G |
| m.960 |  |  | 960.1C |  |  |  |  |  |  |  |  |  |
| m.1243 | T | C | C | C | C |  |  | C | C | C | C | C |
| m.1406 | T | C | C |  |  |  |  |  |  |  |  |  |
| m.1438 | A | G | G | G | G | G | G | G | G | G | G | G |
| m.1700 | T | C |  |  |  |  |  |  |  |  |  |  |
| m.1811 | A | G |  |  |  |  |  |  |  |  |  |  |
| m.1888 | G | A |  |  |  |  |  |  |  |  |  |  |
| m.2706 | A | G | G | G | G |  | G | G | G | G | G | G |
| m.3010 | G | A |  |  |  |  |  |  |  |  |  |  |
| m.3197 | T | C |  |  |  |  |  |  |  |  |  |  |
| m.3505 | A | G | G | G | G |  |  | G | G | G | G | G |
| m.3531 | G | A |  |  |  |  |  |  |  |  |  | A |
| m.4093 | A | G |  |  | G |  |  |  |  |  |  |  |
| m.4363 | T | C |  | C |  |  |  |  | C |  |  |  |
| m.4580 | G | A |  |  |  |  |  |  |  |  |  |  |
| m.4769 | A | G | G | G | G | G | G | G | G | G | G | G |
| m.4833 | A | G |  |  |  |  |  |  |  | G |  |  |
| m.5046 | G | A | A | A | A |  |  | A | A | A | A | A |
| m.5460 | G | A | A | A | A |  |  | A | A | A | A | A |
| m.6528 | C | T |  | T |  |  |  |  | T |  |  |  |
| m.7028 | C | T | T | T | T |  | T | T | T | T | T | T |
| m.7864 | C | T |  |  |  |  |  | T |  | T | T |  |
| m.8251 | G | A | A | A | A |  |  | A | A | A | A | A |
| m.8270 |  |  |  |  |  |  |  |  |  | 8270_8278  DelCACCCCCTC |  |  |
| m.8610 | T | C |  |  | C |  |  |  |  |  |  |  |
| m.8614 | T | C |  |  | C |  |  |  |  |  |  |  |
| m.8860 | A | G | G | G | G | G | G | G | G | G | G | G |
| m.8994 | G | A | A | A | A |  | A | A | A | A | A | A |
| m.9055 | G | A |  |  |  |  |  |  |  |  |  |  |
| m.9123 | G | A |  |  |  |  |  |  |  |  |  |  |
| m.9275 | A | G |  |  |  |  |  |  | G |  |  |  |
| m.9698 | T | C |  |  |  |  |  |  |  |  |  |  |
| m.10097 | A | G |  | G |  |  |  |  | G |  |  |  |
| m.10238 | T | C |  |  |  |  |  |  |  |  |  |  |
| m.10310 | G | A |  |  |  |  |  |  |  |  | A |  |
| m.10410 | T | C |  | C |  |  |  |  | C |  |  |  |
| m.10463 | T | C |  |  |  |  |  |  |  |  |  |  |
| m.10550 | A | G |  |  |  |  |  |  |  |  |  |  |
| m.11227 | C | A |  |  |  |  |  |  |  |  | A |  |
| m.11251 | A | G |  |  |  |  |  |  |  |  |  |  |
| m.11299 | T | C |  |  |  |  |  |  |  |  |  |  |
| m.11467 | A | G |  |  |  |  |  |  |  |  |  |  |
| m.11674 | C | T | T | T | T |  |  | T | T | T | T | T |
| m.11719 | G | A | A | A | A |  |  | A | A | A | A | A |

*Table S6 is continued on the next page*

**Table S6 Coding region variants detected by re-sequencing (Sanger) of complete mtDNA of each five lean and obese individuals – *continued***

|  |  |  | **Individual ^a^ (Haplogroup ^b^)** | | | | | | | | | |
| --- | --- | --- | --- | --- | --- | --- | --- | --- | --- | --- | --- | --- |
| **Position ^c^** | **Reference Allele ^d^** | **Variant Allele** | **1 (W)** | **2 (W)** | **3 (W)** | **4 (H)** | **5 (HV)** | **6 (W)** | **7 (W)** | **8 (W)** | **9 (W)** | **10 (W)** |
| m.11812 | A | G |  |  |  |  |  |  |  |  |  |  |
| m.11914 | G | A |  |  |  |  |  |  |  |  |  |  |
| m.11947 | A | G | G | G | G |  |  | G | G | G | G | G |
| m.12007 | G | A |  |  |  |  |  |  |  |  |  |  |
| m.12308 | A | G |  |  |  |  |  |  |  |  |  |  |
| m.12414 | T | C | C | C | C |  |  | C | C | C | C | C |
| m.12612 | A | G |  |  |  |  |  |  |  |  |  |  |
| m.12705 | C | T | T | T | T |  |  | T | T | T | T | T |
| m.12923 | G | T | T |  |  |  |  |  |  |  |  |  |
| m.13368 | G | A |  |  |  |  |  |  |  |  |  |  |
| m.13617 | T | C |  |  |  |  |  |  |  |  |  |  |
| m.13708 | G | A |  |  |  |  |  |  |  |  |  |  |
| m.14148 | A | G |  |  |  |  |  |  |  |  | G |  |
| m.14470 | T | A |  |  |  | A |  |  |  |  |  |  |
| m.14602 | A | G |  |  |  | G |  |  |  |  |  |  |
| m.14766 | C | T | T | T | T |  |  | T | T | T | T | T |
| m.14905 | G | A |  |  |  |  |  |  |  |  |  |  |
| m.15043 | G | A |  |  |  |  |  |  |  |  |  |  |
| m.15218 | A | G |  |  |  |  |  |  |  |  |  |  |
| m.15326 | A | G | G | G | G | G | G | G | G | G | G | G |
| m.15452 | C | A |  |  |  |  |  |  |  |  |  |  |
| m.15607 | A | G |  |  |  |  |  |  |  |  |  |  |
| m.15775 | A | G |  | G |  |  |  |  | G |  |  |  |
| m.15884 | G | C | C | C | C |  |  | C | C | C | C | C |
| m.15924 | A | G |  |  |  |  |  |  |  |  |  |  |

^a^ Individual 1-5 extremely obese children and adolescents; individual 6-10 lean adults

^b^ Haplogroup determined using Affymetrix Genome-Wide Human SNP Array 6.0 data and HaploGrep (Kloss-Brandstätter et al. 2011) based on Phylotree built 11 (van Oven and Kayser 2009); only individuals with HaploGrep's Quality ≥ 90 % were included

^c^ grey shaded positions represent the 40 SNPs present on the Affymetrix Genome-Wide Human SNP Array 6.0

^d^ according to rCRS (Andrews et al. 1999); only deviations from the reference are shown

References:

Andrews RM, Kubacka I, Chinnery PF, Lightowlers RN, Turnbull DM, et al. (1999) Reanalysis and revision of the Cambridge reference sequence for human mitochondrial DNA. Nat Genet 23(2):147.

Kloss-Brandstätter A, Pacher D, Schönherr S, Weissensteiner H, et al. (2011) HaploGrep: a fast and reliable algorithm for automatic classification of mitochondrial DNA haplogroups. Hum Mutat 32(1):25-32.

van Oven M, Kayser M (2009) Updated comprehensive phylogenetic tree of global human mitochondrial DNA variation. Hum Mutat 30(2):E386-E394. http://www.phylotree.org.
