# Supplementary material for: Mitochondrial DNA Variants in Obesity
Source: PLoS One. 2014 May 2;9(5):e94882. doi: 10.1371/journal.pone.0094882 (PMC4008486; doi:10.1371/journal.pone.0094882)
Supplement: Table S7 — D-loop variants (single nucleotide exchanges) detected by re-sequencing (Sanger) of mtDNA and frequencies in cases and controls. (DOCX) [file pone.0094882.s009.docx]

Table S7 D-loop variants (single nucleotide exchanges) detected by re-sequencing (Sanger) of mtDNA and frequencies in cases and controls

| **Detected variants** | **Frequency cases [%]** | **Frequency controls [%]** | **Detected variants** | **Frequency cases [%]** | **Frequency controls [%]** |
| --- | --- | --- | --- | --- | --- |
|  | **n=191** | **n=191** |  | **n=191** | **n=191** |
| **m.63T/C** | 0.00 | 0.52 | **m.310T/C** | 0.52 | 0.52 |
| **m.64C/T** | 0.52 | 1.05 | **m.316G/A** | 0.52 | 0.00 |
| **m.72T/C** | 4.71 | 2.62 | **m.318T/C** | 0.52 | 0.00 |
| **m.73A/G** | 53.40 | 53.40 | **m.319T/C** | 1.57 | 1.57 |
| **m.73A/T ^a^** | 0.00 | 0.52 | **m.321T/C** | 0.00 | 0.52 |
| **m.93A/G** | 1.57 | 0.52 | **m.340C/T** | 0.52 | 0.00 |
| **m.94G/A** | 0.00 | 0.52 | **m.357A/G** | 0.00 | 0.52 |
| **m.95A/C** | 0.52 | 0.00 | **m.372T/C** | 0.00 | 0.52 |
| **m.114C/T** | 0.00 | 0.52 | **m.385A/G** | 1.57 | 0.00 |
| **m.118G/C ^a^** | 0.00 | 0.52 | **m.408T/A** | 0.52 | 1.05 |
| **m.119T/C** | 0.00 | 1.05 | **m.449T/C** | 0.52 | 0.00 |
| **m.143G/A** | 0.52 | 0.52 | **m.455T/C** | 0.52 | 0.00 |
| **m.146T/C** | 9.95 | 10.21^c^ | **m.456C/T** | 6.28 | 4.71 |
| **m.150C/T** | 9.42 | 7.85 | **m.458C/T** | 0.52 | 1.05 |
| **m.151C/T** | 0.52 | 0.26 ^c^ | **m.462C/T** | 8.90 | 6.28 |
| **m.152T/C** | 25.65 | 23.04 | **m.469C/T** | 0.00 | 0.52 |
| **m.153A/G** | 1.05 | 0.00 | **m.477T/C** | 4.71 | 3.66 |
| **m.182C/T** | 0.00 | 0.52 | **m.480T/C** | 0.00 | 0.52 |
| **m.183A/G** | 0.00 | 0.52 | **m.482T/C** | 0.52 | 0.00 |
| **m.185G/A** | 6.81 | 4.71 | **m.489T/C** | 12.57 | 7.85 |
| **m.188A/G** | 1.57 | 3.14 | **m.497C/T** | 2.09 | 4.19 |
| **m.189A/G** | 0.52 | 3.66 | **m.499G/A** | 5.24 | 3.66 |
| **m.193A/G** | 0.00 | 0.52 | **m.508A/G** | 1.05 | 1.05 |
| **m.194C/T** | 0.52 | 1.57 | **m.509C/T** | 0.52 | 0.00 |
| **m.195T/C** | 18.06 ^c^ | 18.59 ^c^ | **m.513G/A** | 2.09 | 1.57 |
| **m.196T/C** | 0.00 | 0.52 | **m.533A/G** | 0.00 | 1.05 |
| **m.198C/T** | 0.52 | 0.52 | **m.535C/T** | 0.00 | 0.52 |
| **m.199T/C** | 2.62 | 3.14 | **m.549T/C** | 0.26 ^c^ | 0.00 |
| **m.200A/G** | 1.05 | 0.52 | **m.550C/T ^a^** | 0.00 | 0.52 |
| **m.203G/A** | 1.57 | 0.00 | **m.564G/A ^a^** | 0.52 | 0.00 |
| **m.204T/C** | 2.62 | 7.33 | **m.567A/C** | 0.52 | 0.00 |
| **m.207G/A** | 2.62 | 6.28 | **m.568C/T ^a^** | 0.52 | 0.00 |
| **m.210A/G** | 0.52 | 0.00 | **m.574A/C ^a^** | 0.52 | 0.00 |
| **m.215A/G** | 1.57 | 1.57 | **m.628C/A ^a^** | 0.00 | 0.52 |
| **m.217T/C** | 1.05 | 0.52 | **m.634T/C** | 0.00 | 0.52 |
| **m.225G/A** | 1.05 | 0.00 | **m.678T/C** | 0.52 | 0.00 |
| **m.226T/C** | 1.05 | 0.00 | **m.16051A/G** | 2.09 | 2.62 |
| **m.227A/T** | 0.52 | 0.00 | **m.16063T/C** | 0.52 | 0.00 |
| **m.228G/A** | 5.24 | 4.19 | **m.16067C/T** | 0.52 | 0.00 |
| **m.234A/G** | 0.52 | 0.00 | **m.16069C/T** | 11.52 | 7.85 |
| **m.235A/G** | 1.57 | 0.00 | **m.16082C/T** | 0.52 | 0.00 |
| **m.236T/C** | 0.00 | 0.52 | **m.16086T/C** | 1.57 | 1.05 |
| **m.239T/C** | 2.09 | 3.14 | **m.16092T/C** | 1.05 | 0.52 |
| **m.240A/T ^a^** | 0.00 | 0.52 | **m.16093T/C** | 5.76 | 5.24 |
| **m.242C/T** | 1.57 | 2.09 | **m.16104C/T** | 0.00 | 1.05 |
| **m.246T/C** | 0.52 | 0.00 | **m.16104C/A** | 0.52 | 0.00 |
| **m.247G/A** | 1.05 | 0.52 | **m.16111C/T** | 0.00 | 0.52 |
| **m.250T/C** | 2.09 | 2.09 | **m.16114C/T** | 1.05 | 0.26 ^c^ |
| **m.257A/G** | 1.05 | 0.52 | **m.16126T/C** | 23.04 | 19.37 |
| **m.259A/G** | 0.52 | 0.00 | **m.16129G/A** | 4.19 | 5.24 |
| **m.260G/A** | 0.52 | 0.00 | **m.16129G/C** | 1.05 | 0.52 |
| **m.262C/T** | 0.52 | 0.00 | **m.16134C/T** | 0.52 | 2.09 |
| **m.263A/G** | 97.91 | 99.48 | **m.16145G/A** | 4.19 | 3.66 |
| **m.282T/C** | 0.00 | 2.09 | **m.16146A/G** | 0.00 | 1.05 |
| **m.285C/T** | 1.05 | 0.52 | **m.16147C/A** | 0.00 | 0.52 |
| **m.295C/T** | 10.99 | 7.85 | **m.16147C/T** | 0.00 | 0.52 |
| **m.295C/A** | 0.00 | 1.05 | **m.16148C/T** | 0.52 | 0.52 |

*Table S7 is continued on the next page*

**Table S7 D-loop variants (single nucleotide exchanges) detected by re-sequencing (Sanger) of mtDNA and frequencies in cases and controls – *continued***

| **Detected variants** | **Frequency cases [%]** | **Frequency controls [%]** | **Detected variants** | **Frequency cases [%]** | **Frequency controls [%]** |
| --- | --- | --- | --- | --- | --- |
|  | **n=191** | **n=191** |  | **n=191** | **n=191** |
| **m.16153G/A** | 1.05 | 0.00 | **m.16265A/G** | 2.09 | 0.52 |
| **m.16154T/C** | 0.00 | 0.52 | **m.16266C/T** | 1.57 | 1.57 |
| **m.16160A/G** | 0.52 | 0.00 | **m.16269A/G** | 0.52 | 0.00 |
| **m.16162A/G** | 2.62 | 4.71 | **m.16270C/T** | 6.81 | 4.71 |
| **m.16163A/G** | 2.62 | 2.62 | **m.16271T/C** | 0.00 | 1.05 |
| **m.16164A/G** | 0.00 | 0.52 | **m.16274G/A** | 0.52 | 0.00 |
| **m.16168C/T** | 0.52 | 0.00 | **m.16278C/T** | 3.14 | 1.57 |
| **m.16170A/G** | 0.52 | 0.00 | **m.16284A/G** | 0.52 | 0.00 |
| **m.16171A/T** | 0.26 ^c^ | 0.00 | **m.16286C/T** | 0.52 | 0.52 |
| **m.16172T/C** | 4.19 | 3.14 | **m.16288T/C** | 0.52 | 0.00 |
| **m.16174C/T** | 1.05 | 0.52 | **m.16289A/G** | 0.52 | 0.00 |
| **m.16176C/T** | 0.26 ^c^ | 0.52 | **m.16290C/T** | 0.00 | 0.52 |
| **m.16176C/G** | 0.52 | 0.52 | **m.16291C/T** | 2.62 | 0.52 |
| **m.16179C/T** | 0.52 | 0.00 | **m.16292C/T ^b^** | **0.00** | **4.19** |
| **m.16180A/G** | 0.00 | 0.52 | **m.16293A/T** | 0.00 | 0.52 |
| **m.16182A/G** | 0.52 | 0.52 | **m.16293A/G** | 2.09 | 2.62 |
| **m.16182A/C** | 3.14 | 0.52 | **m.16294C/T** | 10.99 | 11.52 |
| **m.16183A/C** | 3.66 | 4.19 | **m.16295C/T** | 0.00 | 0.52 |
| **m.16185C/T** | 0.52 | 0.00 | **m.16296C/T** | 5.24 | 7.33 |
| **m.16186C/T** | 3.14 | 2.09 | **m.16297T/C** | 0.00 | 0.52 ^c^ |
| **m.16187C/T** | 0.00 | 0.52 | **m.16298T/C** | 6.81 | 4.71 ^c^ |
| **m.16188C/A** | 0.00 | 0.52 | **m.16300A/G** | 0.00 | 0.26 |
| **m.16188C/G** | 0.00 | 0.52 | **m.16301C/T** | 0.00 | 0.26 |
| **m.16189T/C ^b^** | **16.75** | **9.42** | **m.16304T/C** | 10.47 | 12.04 ^c^ |
| **m.16192C/T** | 4.71 | 3.14 | **m.16309A/G** | 0.00 | 0.52 |
| **m.16193C/T** | 1.57 | 2.09 | **m.16311T/C** | 13.61 | 15.97 |
| **m.16201C/T** | 0.52 | 0.00 | **m.16316A/G** | 0.00 | 1.05 |
| **m.16209T/C** | 2.62 | 4.19 | **m.16318A/T** | 0.52 | 0.52 |
| **m.16213G/A** | 0.00 | 1.57 | **m.16319G/A** | 0.00 | 0.52 |
| **m.16215A/G** | 0.52 | 0.00 | **m.16320C/T** | 1.05 | 1.57 |
| **m.16216A/G** | 0.52 | 0.00 | **m.16324T/C** | 0.52 | 1.57 |
| **m.16217T/C** | 0.00 | 0.52 | **m.16325T/C** | 0.00 | 1.57 ^c^ |
| **m.16218C/T** | 0.52 | 0.52 | **m.16327C/T** | 0.00 | 0.52 |
| **m.16219A/G** | 0.00 | 1.57 | **m.16335A/G** | 0.00 | 0.79 |
| **m.16221C/T** | 1.05 | 0.52 | **m.16342T/C** | 0.52 | 1.57 |
| **m.16222C/T** | 3.14 | 1.57 | **m.16343A/G** | 0.52 | 1.05 |
| **m.16223C/T** | 5.76 | 7.85 | **m.16344C/T** | 0.52 | 0.00 |
| **m.16224T/C** | 5.24 | 7.85 | **m.16353C/T** | 0.00 | 0.52 |
| **m.16230A/G** | 0.00 | 0.52 | **m.16354C/T** | 0.52 | 0.00 |
| **m.16231T/C** | 1.57 | 1.57 | **m.16355C/T** | 1.57 | 1.57 |
| **m.16234C/T** | 1.05 | 2.09 | **m.16356T/C** | 6.28 | 5.76 |
| **m.16235A/G** | 0.52 | 0.00 | **m.16357T/C** | 0.52 | 0.00 |
| **m.16239C/T** | 0.00 | 0.52 | **m.16360C/T** | 0.00 | 0.52 |
| **m.16240A/G** | 0.00 | 0.52 | **m.16362T/C** | 7.85 | 8.90 |
| **m.16243T/C** | 0.52 | 0.52 | **m.16366C/T** | 0.52 | 1.05 |
| **m.16245C/T** | 0.52 | 1.57 | **m.16390G/A** | 2.09 | 2.62 |
| **m.16247A/G** | 0.00 | 0.52 | **m.16391G/A** | 2.09 | 2.09 ^c^ |
| **m.16248C/T** | 0.52 | 0.52 | **m.16398G/A** | 1.05 | 0.00 |
| **m.16249T/C** | 1.05 | 0.52 | **m.16399A/G** | 3.66 | 2.88 |
| **m.16256C/T** | 4.19 | 3.14 | **m.16482A/G** | 1.05 | 2.62 |
| **m.16258A/C** | 0.52 | 0.00 | **m.16497A/G** | 0.00 | 1.05 |
| **m.16258A/G** | 0.00 | 0.52 | **m.16519T/C** | 66.49 | 70.16 |
| **m.16260C/T** | 0.52 | 0.52 | **m.16526G/A** | 2.62 | 1.05 |
| **m.16261C/T** | 4.19 | 4.19 | **m.16527C/T** | 0.52 | 0.00 |
| **m.16263T/C** | 1.57 | 1.05 |  |  |  |

^a^ variant has not been described previously based on [www.mitomap.org](file:///C:\Users\Nadja\Diss\www.mitomap.org), last edited on Apr 23, 2013 (Ruiz-Pesini et al. 2007), ^b^ nominal p<0.05, Fisher’s exact test, two-sided, ^c^ including on one point heteroplasmy

Reference:

Ruiz-Pesini E, Lott MT, Procaccio V, Poole JC, Brandon MC, et al. (2007) An enhanced MITOMAP with a global mtDNA mutational phylogeny. Nucleic Acids Res 35 (Database issue):D823-D828.
