# Supplementary material for: Mitochondrial DNA Variants in Obesity
Source: PLoS One. 2014 May 2;9(5):e94882. doi: 10.1371/journal.pone.0094882 (PMC4008486; doi:10.1371/journal.pone.0094882)
Supplement: Table S8 — D-loop variants (complex nucleotide exchanges, insertions and deletions) detected by re-sequencing (Sanger) of mtDNA and frequencies in cases and controls. (DOCX) [file pone.0094882.s010.docx]

Table S8 D-loop variants (complex nucleotide exchanges, insertions and deletions) detected by re-sequencing (Sanger) of mtDNA and frequencies in cases and controls

| **Detected**  **Variants** | **Frequency cases [%]** | **Frequency controls [%]** |
| --- | --- | --- |
|  | **n=191** | **n=191** |
| **m.16183A/CC** | 2.62 | 1.05 |
| **m.16183A/CCC ^a^** | 0.52 | 0.00 |
| **m.16189T/CC ^a^** | 1.05 | 2.62 |
| **m.43_44insG ^a^** | 1.05 | 0.00 |
| **m.297_298insC ^a^** | 0.00 | 0.52 |
| **m.309_310insC** | 34.03 | 39.79 |
| **m.309_310insCC** | 14.14 | 12.57 |
| **m.310_311insTC** | 0.52 | 1.57 |
| **m.315_316insC** | 95.29 | 97.91 |
| **m.315_316insCC** | 1.05 | 0.00 |
| **m.315_316insCCC** | 0.52 | 0.00 |
| **m.451_452insT** | 0.52 | 0.52 |
| **m.514_515insAC** | 5.24 | 8.38 |
| **m.514_515insACAC** | 3.14 | 1.05 |
| **m.514_515insACACAC** | 0.52 | 0.52 |
| **m.567_568insC** | 1.05 | 0.00 |
| **m.567_568insCCC** | 0.52 | 1.05 |
| **m.567_568insCCCC** | 0.52 | 0.00 |
| **m.567_568insCCCCC** | 0.52 | 3.14 |
| **m.576_577insCA ^a^** | 0.52 | 0.00 |
| **m.16193_16194insC** | 0.52 | 0.00 |
| **m.310delT** | 1.05 | 0.00 |
| **m.311_313delCCC** | 0.52 | 0.00 |
| **m.498delC** | 2.62 | 1.05 |
| **m.513_514delGC ^a^** | 0.00 | 0.52 |
| **m.515_516delAC** | 10.99 | 10.99 |
| **m.568delC ^a^** | 0.52 | 0.00 |
| **m.16189delT** | 0.52 | 0.00 |
| **m.16257delC** | 0.52 | 0.00 |

^a^ variant has not been described previously based on [www.mitomap.org](file:///C:\Users\Nadja\Diss\www.mitomap.org), last edited on Apr 23, 2013 (Ruiz-Pesini et al. 2007)

Reference:

Ruiz-Pesini E, Lott MT, Procaccio V, Poole JC, Brandon MC, et al. (2007) An enhanced MITOMAP with a global mtDNA mutational phylogeny. Nucleic Acids Res 35 (Database issue):D823-D828.
