# Supplementary material for: Mitochondrial DNA Variants in Obesity
Source: PLoS One. 2014 May 2;9(5):e94882. doi: 10.1371/journal.pone.0094882 (PMC4008486; doi:10.1371/journal.pone.0094882)
Supplement: Table S9 — Distribution of haplogroup frequencies (in%) in study samples compared with West Europeans and Germans. (DOCX) [file pone.0094882.s011.docx]

Table S9 Distribution of haplogroup frequencies (in %) in study samples compared with West Europeans and Germans

| **Haplogroup ^a^** | **CC sample ^b^** | **Trios ^b^** | **KORA ^b, c^** | **SHIP ^b, c^** | **POPGEN ^b, c^** | **West Europeans ^d^** | **Germans ^e^** |
| --- | --- | --- | --- | --- | --- | --- | --- |
| **Number of SNP used for haplogroup determination** | 40 | 35 | 37 | 32 | 35 | - | - |
| **A** | - | - | - | - | 0.6 | - | - |
| **B** | 0.1 | - | 1.9 | - | - | - | - |
| **C** | - | - | - | - | - | - | - |
| **D** | - | - | 0.1 | <0.05 | 0.1 | 1 | 0.6 |
| **F** | - | - | - | - | - | 0 | - |
| **G** | - | - | - | - | - | - | - |
| **H** | 44.4 | 44.5 | 41.6 | 41.2 | 41.2 | 41 | 48.6 ^f^ |
| **I** | - | - | - | - | - | 2 | 1.8 |
| **J** | 10.6 | 8.4 | 8.9 | 9.6 | 11.1 | 9 | 8.4 |
| **K** | 6.5 | 6.2 | 6.3 | 0.6 | 7.8 | 5 | 7.5 |
| **L** | - | 0.1 | 0.1 | <0.05 | 0.1 | 1 | 1.2 |
| **M** | 1.0 | 0.6 | 0.7 | 0.4 | 0.2 | 1 | - ^g^ |
| **N** | 2.6 | 3.5 | 2.5 | 3.3 | 3.0 | 1 | 0.6 |
| **P** | 0.1 | - | - | <0.05 | - | - | - |
| **R** | 0.1 | 0.6 | 0.3 | 0.3 | 0.3 | 0 | 0.3 |
| **S** | - | - | - | - | 0.1 | - | - |
| **T** | 9.3 | 9.8 | 11.2 | 10.6 | 11.9 | 8 | 9.0 |
| **U** | 14.4 | 16.3 | 16.2 | 22.2 | 15.5 | 18 | 13.5 |
| **V** | 3.4 | 3.3 | 3.3 | 3.2 | 2.6 | 7 | 4.5 |
| **W** | 1.7 | 1.4 | 1.9 | 2.2 | 2.2 | 2 | 2.7 |
| **X** | 1.5 | 2.6 | 0.4 | 1.5 | 0.4 | 2 | 1.2 |
| **Z** | - | - | - | <0.05 | - | 0 | - |
| **n. d.** | 4.3 | 2.7 | 4.8 | 4.8 | 2.8 | 3 | - |

^a^ Haplogroup determined using Affymetrix Genome-Wide Human SNP Array 6.0 data and HaploGrep (Kloss-Brandstätter et al. 2011) based on Phylotree built 11 (van Oven and Kayser 2009)

^b^ n. d., not defined, i.e. all individuals with a HaploGrep’s quality <90 %

^c^ whole population-based sample

^d^ Estimations based on means from published frequencies (bearing in mind that sometimes not all haplogroups have been typed), compiled for Mitomap in 2009 ([www.mitomap.org](http://www.mitomap.org), Ruiz-Pesini et al. 2007) only for illustrative purpose

^e^ data based on a total of n=333 German individuals (Pliss et al. 2006)

^f^ including haplogroups HV and preHV

^g^ all individuals of haplogroup M belonged to haplogroup D which directly branches off of M

References:

Kloss-Brandstätter A, Pacher D, Schönherr S, Weissensteiner H, et al. (2011) HaploGrep: a fast and reliable algorithm for automatic classification of mitochondrial DNA haplogroups. Hum Mutat 32(1):25-32.

Pliss L, Tambets K, Loogväli EL, Pronina N, Lazdins M, et al. (2006) Mitochondrial DNA portrait of Latvians: towards the understanding of the genetic structure of Baltic-speaking populations. Ann Hum Genet 70(Pt 4):439-58.

Ruiz-Pesini E, Lott MT, Procaccio V, Poole JC, Brandon MC, et al. (2007) An enhanced MITOMAP with a global mtDNA mutational phylogeny. Nucleic Acids Res 35 (Database issue):D823-D828.

van Oven M, Kayser M (2009) Updated comprehensive phylogenetic tree of global human mitochondrial DNA variation. Hum Mutat 30(2):E386-E394. http://www.phylotree.org.
